# Epidemiological Profile of Patients with Vulvovaginal Candidiasis from a Sexually Transmitted Infection Clinic in Southern Spain

**DOI:** 10.3390/pathogens12060756

**Published:** 2023-05-24

**Authors:** Encarnación Martínez-García, Juan Carlos Martínez-Martínez, Adelina Martín-Salvador, Alberto González-García, María Ángeles Pérez-Morente, María Adelaida Álvarez-Serrano, Inmaculada García-García

**Affiliations:** 1 Guadix High Resolution Hospital, Andalusian Health Service, 18500 Granada, Spain; 2 Department of Nursing, Faculty of Health Sciences, University of Granada, 18016 Granada, Spain; 3 Virgen de las Nieves University Hospital, Andalusian Health Service, 18014 Granada, Spain; 4 Department of Nursing, Faculty of Health Sciences, University of Jaén, 23071 Jaen, Spain; 5 Department of Nursing, Faculty of Health Sciences of Ceuta, University of Granada, 51001 Ceuta, Spain

**Keywords:** vulvovaginal candidiasis, prevalence, epidemiology

## Abstract

Epidemiological data on women suffering from vulvovaginal candidiasis and its recurrence are outdated and vague. The aim of this study was to identify the prevalence of women diagnosed with vulvovaginal candidiasis, as well as the epidemiological profile and associated risk factors in the province of Granada (Spain). Data from the Centre for Sexually Transmitted Infections of the Granada province between 2000 and 2018 (N = 438) were used in this study. Associations between sociodemographic and sexual behaviour variables with vulvovaginal candidiasis were analysed using the Chi-square test and bivariate logistic regression. The prevalence of candidiasis was 14.6%. The sociodemographic profile corresponded to a woman aged 25.14 ± 4.8 years on average, who is of Spanish nationality (60.9%), a student (55.7%), in non-active employment (59.7%), with a higher education (56.7%), single (93.5%), and under 30 years of age (79.7%). Variables associated with this diagnosis were the absence of oro-genital contact (OR = 1.99; 95% CI = 0.25–0.74), having a regular partner (OR = 1.99; 95% CI = 1.05–3.75), and age of sexual debut, with the probability increasing by 12% (95% CI = 1.00–1.24) with each year. In this context, vulvovaginal candidiasis infection is common, and its epidemiological profile is contradictory, so our results do not suggest a relevant role of sexual risk behaviours in the diagnosis. Further research is needed to improve the estimates and factors associated with this infection.

## 1. Introduction

Vulvovaginal candidiasis (VVC) is a prevalent infection of the genitourinary tract, and is a frequent cause of vaginitis [1,2]. It has traditionally been claimed to affect 70–75% of women of reproductive age, with at least one episode in their lifetime, but empirical studies to corroborate this are still lacking [3,4]. In the normal vaginal flora of healthy women there are *Candida* species that can cause VVC, with the most common pathogen being *Candida albicans*. However, other non-albicans *Candida* species also cause symptoms, and are often the cause of recurrent vulvovaginal candidiasis (RVVC), occurring at least four episodes per year [2,5,6]. Although women of all ages, ethnicities, and social strata suffer from this infection [7], not all will develop it later in life, due to multiple and non-specific factors [2,8]. Furthermore, episodes of candidiasis cannot be attributed to a specific trigger, but several extrinsic and intrinsic host virulence factors have been identified as the key contributing factors. Intrinsic factors include genetic, immunological, hormonal, metabolic, hygienic, antibiotic/corticosteroid use, and lifestyle-related factors [1,6,7]. Sexual practices also appear to play a significant role, with epidemiological evidence suggesting that the frequency/periodicity of sexual intercourse, and anogenital and especially oro-genital contact, favour transmission of this yeast species [1,9]. Furthermore, sexual partners of women with VVC may become infected through coital, oral, or anal intercourse, suggesting a potential contribution of this infection to an increased susceptibility to HIV, and vice versa [5,10,11,12]. Furthermore, although the search for new therapeutic strategies is an ever-expanding field of research, effective treatments are not always available [13,14,15]. Thus, despite the remarkable advances in scientific knowledge on CVR, there is still some controversy regarding the reliability of the documented prevalence and recurrences [4]; with the identification of the mechanisms of progression from *Candida* species colonisation to infection [5,7,8]; with the roles of the relevant risk factors, and the contribution of sexual transmission to its pathogenesis [1,16]; or about the most effective treatment measures [5,13,14,15].

Several researchers have stated that VVC/RVVC is a major health problem in the West due to the significant morbidity in women’s lives and its high economic cost [2,5,17]. Moreover, Denning et al. [2] predict an upward trend until 2030, especially in CVR, and particularly in developed countries, even though it is considered as a “neglected disease” in scientific research [7]. Thus, epidemiological data on women with the disease are often outdated and relatively vague, as studies are often either imprecise or have been conducted in populations that are not representative of specific geographical settings [1,7,18]. The aim of this study was to estimate the prevalence of women diagnosed with VVC, and to identify the epidemiological profile and risk factors in the province of Granada, southern Spain.

## 2. Materials and Methods

A retrospective case study was conducted of the records of patients seeking care at the Reference Centre for Sexually Transmitted and Sexually Oriented Diseases in Granada, southern Spain, between the years of 2000 and 2018 (inclusive). This centre offers a universal, free, and confidential medical and counselling service for the diagnosis and management of sexually transmitted infections (STIs) and HIV. It is attached to the Andalusian Health Service and serves a population of about 480,000 inhabitants [19], 65% of whom are women aged 15–64 years [20]. People can either self-refer to this centre or be referred by their physicians from primary care centres or hospital emergency departments. During the time analysed, the centre oversaw more than 28,000 consultations registered in a database containing the medical history of the patients. This history was systematically filled in by the doctors and includes socio-demographic variables, clinical signs and symptoms, results of diagnostic tests, therapeutic evolution, and diagnoses. A sampling procedure described elsewhere [21] was applied to this large database, resulting in the generation of a sample encompassing 1828 cases of people over 18 years of age without cognitive deficit. From these cases, non-pregnant women with a confirmed diagnosis were selected. Two groups were subsequently formed, one with women diagnosed with VVC (*n* = 64), and the other group containing those with other STI diagnoses (*n* = 374). Diagnosis of candidiasis was based on clinical presentations of vaginal discharge with or without microscopy or culture, along with symptomatology.

### 2.1. Statistical Analysis

Descriptive statistics were applied to all our data, using absolute numbers and percentages for qualitative variables, and measures of central tendency (mean and standard deviation) for quantitative variables. The Chi-square test was performed to explore the associations between variables and diagnosis of candidiasis or other STI diagnoses, with the level of significance set at *p* < 0.05. To determine the magnitude of the associations found with the diagnosis of candidiasis, a bivariate logistic regression analysis was applied through calculating crude odds ratios (cOR) and 95% confidence intervals (95% CI). Analyses were performed using IBM SPSS Statistics version 28 (SPSS, Inc., Chicago, IL, USA).

### 2.2. Ethical Considerations

The processing of personal data in this research followed the Organic Law 3/2018 of 5 December on the Protection of Personal Data and Guarantee of Digital Rights. The database for the study was anonymised, so that under no circumstances could individuals be identified. The protocol obtained a favourable resolution from the Biomedical Research Ethics Committee of the province of Granada (code ITS 2018/1766-N-18), and from the Management Directorate of the Granada-Metropolitan Health District, who are responsible for the ITS centre where the research was conducted in November 2018.

## 3. Results

A total of 438 women with at least one confirmed diagnosis were counted in the selected sample. Table 1 shows the main types of diagnoses that were identified, of which 14.6% were VVC.

The socio-demographic variables are shown in Table 2. Infections have been classified into VVC as one category, with the other STI diagnoses placed into a second, separate category. STI diagnoses include human papillomavirus (genital condyloma and cervical human papillomavirus), chlamydia, bacterial vaginosis, molluscum contagiosum, syphilis, genital herpes, gonorrhoea, and other STI infections, as mentioned in Table 1. The mean age of the total sample (standard deviation) was 26.74 ± 7.2 years. The socio-demographic profile of the women diagnosed with candidiasis corresponds to women of Spanish nationality (60.9%), who are students (55.7%), without an active profession (59.7%), with a higher education (56.7%), single (93.5%), and with a mean age (standard deviation) of 25.14 ± 4.8 years. The relationship with profession was determined to be statistically significant, meaning that being a student, professional, or former sex professional was found to be more frequent in women diagnosed with VVC. Professionals or former sex workers/professionals are those women who have been or are in prostitution.

Table 3 shows the risk factors related to sexual behaviour according to diagnostic groups. Having a regular partner (76.7%) was found to be more frequent among women diagnosed with VVC, as well as a higher age of sexual debut (18.23 years vs. 17.29 years, respectively). On the other hand, more women in the group with another STI diagnosis (62.8%) reported engaging in oral-genital sex. The rest of the variables analysed were found to have behaved similarly in both groups.

The binary logistic regression model reveals that having oro-genital contact (cOR = 0.43; 95% CI = 0.25–0.74) decreases the risk of having candidiasis versus another STI diagnosis, whereas having a regular partner increases the risk (cOR = 1.99, 95% CI = 1.05–3.75). For every year increase in age at first intercourse, the probability of having a diagnosis of candidiasis increases by 12% (cOR = 1.12; 95% CI = 1.00–1.24). When dichotomising the variable profession, the previously observed association is lost (Table 4).

## 4. Discussion

This study has determined the prevalence of women with candidiasis in a specific area of the province of Granada (Spain) and has also identified its epidemiological profile. It provides data on an infection for which there are usually no accurate national or local data, and the magnitude of associations of the socio-demographic and sexual behavioural variables with VVC has been explored and quantified. To our knowledge, it is one of the few studies that has been developed in our context with similar objectives [22,23,24,25].

The observed prevalence of candidiasis was between previously documented figures for countries in our environment such as Greece (11–12%) [26,27] or Italy (18–19%) [28,29]. Globally, prevalence rates ranging from 5% to 78% have been reported [18,30,31,32,33,34,35], depending on the study design, geographical location, characteristics of the population analysed, and the diagnostic methods used. This is the reason for which Sobel [36] states that, at present, it is impossible to conduct epidemiological research to know, with any reliability, the estimates, and trends of *Candida* infections. However, the efforts being made to obtain solid data to understand the current burden of this disease and its associated factors, especially at the local level [5,30,36], would be justified by the distress that this infection causes in many affected women, and the number of consultations it generates in the healthcare system [7,37].

Although the registers did not allow differentiation between VVC and RVVC, it was not excluded that most cases were RVVC. Symptoms of VVC are usually mild, and most episodes of symptomatic disease appear as sporadic attacks that many women suffer in silence, and are combatted either through accessing a variety of over-the-counter antifungals, or are managed empirically by their physicians without diagnostic confirmation with microbiological cultures [5,13,18,36,38]. It would therefore be reasonable to assume that the women who have sought care at this specialised STI centre are not seeking care for an acute episode, but rather for complicated, treatment-resistant conditions, or that they have certain risk behaviours for STIs, a question that requires further investigation. In this case, the figure of 14.6% observed was among the highest documented for RVVC, which typically ranges between 5 and 20% [4,17,26,32,39], which would be consistent with both the age of the women diagnosed with candidiasis and the context analysed.

In general, the groups of women compared in this study did not differ substantially either in socio-demographic variables or in their sexual behaviour. The sample analysed not only shared a common geographical area, and thus social and demographic characteristics, but it was also plausible to consider that they may share certain aspects of symptomatology and risk factors, as the symptoms of VVC are similar to those observed of sexually transmitted infections [40]. The difficulty in predicting VVC in terms of risk factors, due to, among other causes, its multifactorial pathogenesis [1,16], together with the common origin of the population analysed, may have therefore biased the study results towards null findings, and explain the modest odds ratios found. However, despite being a similar population group, several epidemiological characteristics were distinct, as Sasani et al. [40] found in several studies.

The socio-demographic profile of women diagnosed with VVC was found to correspond to a Spanish woman, who is a student, not in active employment, with a higher education, who is single, and under 30 years of age. A study conducted in Madrid reported more positive cultures for VVC in foreign women (28.5%) than in Spanish women (25.7%) [22], while López-Olmos et al. [41] in Valencia reported the opposite (16.03% vs. 23.97%). As in our study, in none of these studies was the country of birth found to be statistically associated with infection.

The highest number of patients with VVC was identified as the age group of women under 30 years of age in their reproductive years, which is considered a risk factor [27,28,29,32,42]. This has been attributed to sexual activity and the increased amount of oestrogens produced in this age group that promotes yeast adhesion and penetration into the vaginal mucosa [7,26]. However, our results are consistent with several other studies who did not detect a differential influence of chronological age with the occurrence of VVC [30,43,44,45,46].

In relation to profession, female students and female sex workers/professionals were found to be more frequently diagnosed with VVC, which may be associated with both the culture of the country and the age of the women, as well as exposure to sex. A few studies have reported a higher prevalence of VVC in sex workers [41,47], although other studies found no distinct behaviours among sex workers compared to other groups [48,49]. Although the literature frequently points out that a greater number of sexual relations predisposes to acute vaginitis [1,9,18], there are also authors who do not consider this to be a predisposing factor for fungal colonisation, but rather immunological factors and host resistance [50]. In fact, although it has been shown, for example, that STI acquisition is more frequent among female sex workers, it does not seem to be related to the number of clients or the duration of sex work, but to other factors such as intravenous drug use, condom use, or the number of occasional non-paying clients [47]. This controversial finding may therefore be due to the context of the study, wherein female sex workers are over-represented.

Regarding occupation, the highest number of VVC diagnoses was identified among women who reported that they were not in employment. Foxman et al. [17], in their large study conducted in five European countries and the USA, reported a significantly lower frequency of VVC for women in Spain (*n* = 1002) who did not report an occupation. These differences may be due to the age profile of our study population, as well as the sample size itself, which is an issue that requires further evaluation in additional research. Regarding educational level, we observed more VVC diagnoses in women with higher education, although this variable does not seem to exert a significant influence on the development of infection. However, Benedict et al. [30] reported in a US study of 1869 women, with more than half of whom had college or a higher education equivalent, that the highest risk of having an episode of VVC in the past year was found in those with less than a high school education (OR= 6.30, 95% CI= 1.84–21.65).

Of the sexual behaviours that have been analysed, having had oro-genital contact, having a regular partner, and the age of sexual debut were found to have influenced the diagnosis of VVC. Several studies have reported an association between receptive oro-genital sex and the development of VVC, particularly reinfection, which appears to vary between cultures and ethnic groups [9,17,18,48,51]. However, our findings do not suggest a positive association, but rather go in the opposite direction, as also reported by Bardin et al. for women with vulvovaginal disorders such as VVC [52]. Having a stable partner was more frequent among women diagnosed with VVC, as has been documented by other researchers [30,33], an association which, moreover, in our work reached statistical significance. This result could be due to not only to the fact that VVC is not considered as an STI, but also to the higher representation in our sample of women who reported living with a partner. Also, later sexual debut increased the likelihood of VVC, as previously reported [4]. Thus, although the role of sexual behaviours in causing often recurrent VVC has been underestimated [1], our results are in general agreement with the epidemiological studies that do not suggest such an association [4,48,52].

This study has certain strengths and limitations that need to be highlighted. As strengths, it is worth noting that access was gained to the records of a reference health centre for a local setting, with varied and individualised information, and over a considerable period. This has provided a robust dataset from routine clinical practice, exploring the associations between individual variables, and quantifying their magnitude in relation to the diagnosis of VVC. In addition, the clinical nature of the diagnosis of candidiasis is a strength of this study. Self-report of medical diagnoses by women is often one of the main limitations of research attempting to estimate prevalence and recurrences at a population level, as they tend to over-diagnose [4,17,18,32,36]. However, although the diagnosis has been attempted to be confirmed with conventional laboratory tests, it has been widely documented that clinicians both over-diagnose and underdiagnose erroneously, implying a classification bias. The non-specific clinical presentation of signs and symptoms of this infection, the current clinical approaches too often based on empiricism and trial and error, and the fact that the physicians at the STI Centre were not gynaecologists, are potential concerns that need to be considered [3,4,30,32,36].

An existing database not specifically designed for the analysis of VVC/RVVC cases has been exploited, and therefore, as a secondary source of information, has presented data gaps that were necessary to have been identified for a more accurate epidemiological profile. Blostein et al. [32] have already pointed out that all existing databases are subjected to, among other limitations, diagnostic vagaries, and therefore present major challenges for the accurate quantification of VVC burden. Nevertheless, the observed prevalence is among those documented in neighbouring countries, but we recognise that it has undoubtably been underestimated, due to the selection bias generated by a very restrictive sample of symptomatic women with a confirmed diagnosis attending an STI diagnosis and treatment centre. Thus, not only have we missed an undetermined number of cases by not including asymptomatic patients, estimated in the literature to be between 6% and 17% [1,7,9,29], but also the ability to have detected more significant and larger associations, as asymptomatic women tend to have fewer risky sexual practices [48]. Therefore, our results are not generalizable to the rest of the women with VVC/RVVC who attend other types of health centres. Finally, although the cross-sectional design of the study does not allow us to establish causal relationships, our findings raise hypotheses that may serve as a useful resource for future prospective and multicentre research.

## 5. Conclusions

VVC infection is frequent in our context, and its epidemiological profile is contradictory, meaning our results do not suggest a relevant role of sexual risk behaviours in the diagnosis of VVC. The data from this study represent a body of information on VVC in the province of Granada (Spain) that may serve as a useful resource for future research related to this infection. We note that the prevalence of VVC, although it may be suspected to be underestimated, is a common condition also in this context, and similar to those documented for surrounding countries. These results support the research that does not link risky sexual practices to the development of infection, but the epidemiological data remains controversial. Despite the difficulty in making a reliable estimate of this infection and its associated risk factors, we encourage the scientific community to continue their research on this condition of increasing incidence worldwide, with the aim of proposing prevention strategies, acting on potentially reversible determinants, and improving health outcomes for affected patients.

## Figures and Tables

**Table 1:** Diagnoses in women attending the Centre for Sexually Transmitted Infections (Granada). N = 438.

	*n*	%
Human papillomavirus	96	21.9
Vulvovaginal candidiasis	64	14.6
Chlamydiasis	34	7.8
Bacterial vaginosis	23	5.2
Molluscum contagiosum	21	4.8
Syphilis	11	2.5
Genital herpes	9	2.1
Gonorrhoea	6	1.4
Other	174	39.7

**Table 2:** Socio-demographic variables in women diagnosed with VVC or other STI diagnoses.

	Candidiasis		Other STI diagnoses		Total		*p*-Value
	*n*	%	*n*	%	*n*	%	
Nationality (*n* = 436)							
Spanish	39	60.9	264	71.0	303	69.5	0.107
Foreign	25	39.1	108	29.0	133	30.5	
Profession (*n* = 414)							
Prof./ex prof. Sex	17	27.9	69	19.5	86	20.8	0.002
Student	34	55.7	144	40.8	150	36.2	
Other	10	16.4	140	39.7	178	43.0	
Employment status (*n* = 392)							
Active	25	40.3	146	44.2	171	43.6	0.568
Inactive	37	59.7	184	55.8	221	56.4	
Educational level (*n* = 418)							
Superiors	34	56.7	186	52	220	52.6	0.499
Other	26	43.3	172	48	198	47.4	
Marital status (*n* = 431)							
Single	58	93.5	312	84.6	370	85.8	0.060
Other	4	6.5	57	15.4	61	14.2	
Age (*n* = 438)							
(Range) Mean ± SD	(16–37) 25.14 ± 4.8		(16–60) 27.01 ± 7.5		(16–60) 26.74 ± 7.2		0.057
<30 years	51	79.7	271	72.5	322	73.5	0.226
≥30 years	13	20.3	103	27.5	116	26.5	

Abbreviations: SD, standard deviation; and *p*, *p*-value.

**Table 3:** Sexual behaviours in women diagnosed with VVC or other STI diagnoses.

	Candidiasis		Other STI diagnosis		Total		*p*-Value
	*n*	%	*n*	%	*n*	%	
Sexual orientation (*n* = 422)							
Straight	59	98.3	338	93.4	397	94.1	0.131
Another	1	1.7	24	6.6	25	5.9	
Usual partner (*n* = 410)							
Yes	46	76.7	218	62.3	264	64.4	0.032
No	14	23.3	132	37.7	146	35.6	
Couple with symptoms (*n* = 169)							
Yes	7	26.7	50	35.0	57	33.7	0.425
No	19	73.1	93	65.0	112	66.3	
Oro-genital contact (*n* = 438)							
Yes	27	42.2	235	62.8	262	59.8	0.002
No	37	57.8	139	37.2	176	40.2	
Anogenital contact (*n* = 438)							
Yes	15	23.4	87	23.3	102	23.3	0.976
No	49	76.6	287	76.7	336	76.7	
Number of lifetime sexual partners (*n* = 140)							
≤10	8	57.1	81	64.3	89	63.6	0.598
>10	6	42.9	45	35.7	51	36.4	
STI history (*n* = 372)							
Yes	15	28.3	80	25.1	95	25.5	0.618
No	38	71.7	239	74.9	277	74.5	
Drug use (*n* = 260)							
Yes	6	21.4	71	30.6	77	29.6	0.315
No	22	78.6	161	69.4	183	70.4	
Age at first sexual intercourse (*n* = 328)							
(Range) Mean ± SD	(13–30) 18.23 ± 3.6		(13–28) 17.29 ± 2.4		(13–30) 17.42 ± 2.6		0.032

Abbreviations: SD, standard deviation; and *p*, *p*-value.

**Table 4:** Bivariate logistic regression for candidiasis.

	cOR	95% CI
Profession		
Ex./Sex professional	0.62	0.33–1.16
Another	1	
Usual partner		
Yes	1.99	1.05–3.75
No	1	
Oro-genital contact		
Yes	0.43	0.25–0.74
No	1	
Age at first sexual intercourse	1.12	1.00–1.24

Abbreviations: cOR, crude odds ratio; and CI, confidence interval.

## Data Availability

Data are contained within the article.
